# Distribution of Country of Origin in Studies Used in Cochrane Reviews

**DOI:** 10.1371/journal.pone.0018798

**Published:** 2011-04-19

**Authors:** Robert F. Wolff, Stefan Reinders, Michael Barth, Gerd Antes

**Affiliations:** 1 Institute of Medical Biometry and Medical Informatics, German Cochrane Centre, Department of Medical Biometry and Statistics, University Hospital Freiburg, Freiburg, Germany; 2 Faculty of Health Sciences, Bielefeld School of Public Health, Bielefeld, Germany; 3 Institut für Medizinische Informationsverarbeitung, Biometrie und Epidemiologie, Ludwig Maximilian University of Munich, Munich, Germany; Central Institute of Educational Technology, Canada

## Abstract

**Background and Objective:**

Inclusion in systematic reviews is one important component in judging the potential impact of clinical studies upon practice and hence the ‘value for money’ of spending for clinical research. This study aims to quantify the distribution of countries of origin of clinical studies used in Cochrane Reviews (CRs), and to link these data to the size of a country and to its spending on research.

**Methods:**

Random sample of publications used for CRs published in Issue 1 2008 and of publications used in CRs in the field of complementary and alternative medicine (CAM). Publications without original data were excluded. Likely countries of origin determined based on abstracts/full texts. CIA World Factbook (population data) and OECD database (economic data) were used.

**Results:**

1,000 random entries out of 140,005 references available in all specialities. In 876 (91.4%) of 959 eligible studies, country of origin was determined. The USA was the leading contributor (36.0% of the studies), followed by UK (13.4%), Canada (5.3%), Australia and Sweden (3.7%). In the CAM sample, country of origin was determined in 458 (93.5%) of 497 assessed studies. Again, the USA was the leading contributor (24.9%), with China also emerging as a significant contributor (24.7%) in this field. For both samples, the contribution of smaller countries (especially Scandinavian countries, Greece, and Ireland) became more noteworthy when considered in relation to population size and research spending.

**Conclusions:**

Our results support the leading roles of both the USA and the UK in publishing clinical papers. The emerging role of China can be seen, particularly related to CAM studies. Taking into account size of population and economic power, countries like France, Germany, Italy, and Spain provide small contributions. In contrast, smaller countries like Australia, Denmark, Finland, Ireland, New Zealand, and Sweden also play major roles.

## Introduction

Back in 1747, the Scottish naval surgeon James Lind conducted one of the first controlled clinical trials (CCT) [1]. Since the end of the Second World War hundreds of thousands of CCTs and randomised controlled trials (RCTs) have been conducted all over the world [2], [3]. Today, clinical trials can be seen as the backbone of systematic reviews [4]. Systematic reviews have a decisive role in clinical decision making [5], [6].

Studies assessing the geographical distribution of clinical research activity have confirmed the leading role of the USA in publishing scientific papers in various fields: in the top 50 biomedical journals [7], clinical cardiology [8], clinical radiology [9], clinical oncology [10], drug trials [11] and biomedical research [12]. In addition, various publications on the contribution of countries to publications of specific journals are available [13], [14].

To date, we are aware of only one study that has examined the production of RCTs and CCTs per country across all specialities and journals [15]. The authors of this study used “Clinical Trials”, formerly known as “Cochrane Central Register on Controlled Trials” (CENTRAL), in the Cochrane Library to create a ranking of countries with respect to the numbers of published RCTs and CCTs [16]. In addition, they tried to assess the relationship between the number of inhabitants per country and publication rates by the performance of an ecological study.

Based on this concept of Gluud and Nikolova [15], we have evaluated the studies used for systematic reviews published by the Cochrane Collaboration. The “Cochrane Database of Systematic Reviews” (“Cochrane Reviews”) as part of the Cochrane Library contains 3,372 reviews and 1,776 protocols for reviews (Issue 1 2008).

As thorough searches are conducted for Cochrane Reviews (including handseaching and searches for non-English studies), they are likely to include a high proportion of the available studies in any clinical field [3]. Usage of a clinical study in systematic reviews can be used as a proxy for quality and the practical value of the trial. Systematic reviews and hence the studies included in them form the evidence body supporting any clinical guidance, such as guidelines, evidence-based patient information and websites, and reimbursement decisions (health technology assessments).

Our study aims to determine the contribution of clinical studies per country across all specialties and to examine the production of clinical studies in the field of complementary and alternative medicine (CAM). We have also assessed the relationships between the contribution rate and the population size and spending on research and development of each country.

## Methods

### Literature search

The database of studies used for Cochrane Reviews published in Issue 1 2008 of the Cochrane Database of Systematic Reviews was used as a source of data. This database includes all studies retrieved for Cochrane Reviews, those which were finally included in reviews, those which were excluded as well as cited publications. The sample of CAM related studies was created from a selection of Cochrane Reviews, identified as CAM related reviews by the complementary and alternative medicine field of the Cochrane Collaboration [17].

All stages of study selection and data extraction were done by one of three reviewers (RW, SR or MB) and checked independently by a second reviewer (RW, SR or MB). Any disagreement during the selection, extraction, and assessment process was resolved by discussion and consensus.

### Study selection

Samples of studies were drawn using the “SURVEYSELECT” command in SAS 9.1.3 Service Pack 4 and SPSS for Windows 11.5.1.

Study samples were screened for fulfilling the inclusion criteria. Eligible studies were included in the process of assessing the country of origin.

### Inclusion criteria

Publications assessed for inclusion in Cochrane Reviews were included. In a first step, we excluded studies awaiting assessment or marked as ongoing trials. Secondly, we excluded reviews, studies focussing on economical or methodological aspects, publications without original data (like editorials, comments, letters to the editor), studies on animals and studies without reference.

### Data extraction and quality assessment

The abstract and/or full text of each included study was checked to determine the likely country of origin. The decision was based on participating centres and hospitals, the responsible ethics committee, the funding source, and the affiliation of the authors. Cases in which the likely country of origin could not be determined were excluded; this included multicentre studies conducted in various countries where no lead country could be identified. Accordingly, studies with a clearly identifiable leading country were only counted once.

Various sources were used to gather information about the studies: original publications, data from trial registries, Google Scholar, ISI Web of Knowledge, and the websites of the journals publishing included studies. If necessary, we contacted the authors of related Cochrane Reviews to obtain further information.

The CIA World Factbook [18] was used to extract data on population of countries. The economic data were drawn from the OECD Statistics Portal of the Organisation for Economic Co-operation and Development (OECD) [19]. We calculated the spending on research and development (R&D) using the Gross Domestic Product (GDP) and the percentage spent on R&D.

### Data analysis

We used Microsoft® Office Excel 2007 for data analysis and for presentation of the data.

## Results

### Description of search and selection process

**Figure 1:**
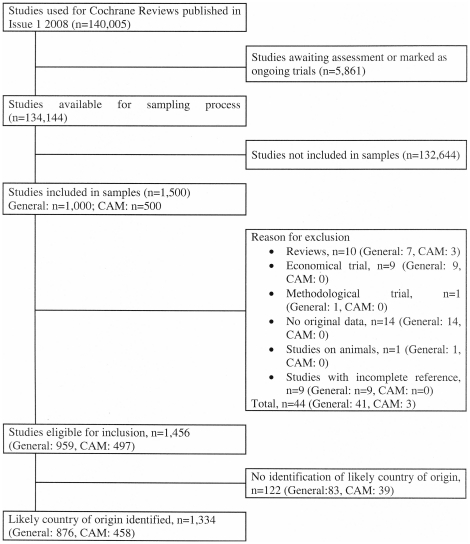
Flow diagram according to the QUOROM-statement [27] with the total number of studies used for Cochrane Reviews and the number included in the present study. *CAM = Complementary and alternative medicine.*

The Cochrane Reviews published in Issue 1 2008 contained 140,005 references to studies. After exclusion of studies awaiting assessment and ongoing studies, 134,144 were available for assessment. From this data set, we randomly sampled 1,000 studies across all specialities and 500 CAM-related studies. After checking for studies meeting the exclusion criteria, 959 and 497 studies respectively were available for data assessment (Figure 1).

### Country of origin

We were able to identify the likely country of origin of 876 of 959 studies across all specialities (91.3%), and 458 of 497 CAM-related studies (92.2%).

Across all specialities, the USA published most CCTs and RCTs (n = 315 of 876, 36.0%), followed by the United Kingdom (UK, 117, 13.4%), Canada (46, 5.3%), Australia (32, 3.7%), and Sweden (32, 3.7%).

When only CAM-related studies were considered, the USA remained the country with the highest number of publications (n = 114 of 458, 24.9%), followed by China (113, 24.7%), the UK (59, 12.9%), Germany (17, 3.7%) and Italy (13, 2.8%). These results are presented in Table 1.

**Table 1:** Table showing the first 30 entries of likely country of origin for studies within all specialities (left) and CAM-related studies (right).

Studies within all specialities			CAM-related studies		
Country	n =	%	Country	n =	%
USA	315	36.0	USA	114	24.9
United Kingdom	117	13.4	China	113	24.7
Canada	46	5.3	United Kingdom	59	12.9
Australia	32	3.7	Germany	17	3.7
Sweden	32	3.7	Italy	13	2.8
Germany	31	3.5	Denmark	11	2.4
Italy	30	3.4	Netherlands	10	2.2
Netherlands	25	2.9	Australia	10	2.2
France	24	2.7	Canada	10	2.2
Denmark	21	2.4	Japan	10	2.2
Japan	19	2.2	India	9	2.0
China	17	1.9	Sweden	8	1.7
Finland	17	1.9	Finland	6	1.3
India	14	1.6	France	6	1.3
Spain	12	1.4	Israel	5	1.1
Israel	10	1.1	Hungary	5	1.1
Switzerland	8	0.9	Norway	4	0.9
Belgium	7	0.8	Taiwan	4	0.9
Ireland	7	0.8	Poland	4	0.9
Austria	6	0.7	Switzerland	3	0.7
Norway	6	0.7	Romania	3	0.7
South Africa	6	0.7	Spain	3	0.7
Greece	5	0.6	South Africa	3	0.7
Mexico	5	0.6	Nigeria	3	0.7
Turkey	5	0.6	Brazil	3	0.7
Brazil	3	0.3	New Zealand	2	0.4
Czech Republic	3	0.3	Austria	2	0.4
Hungary	3	0.3	Belgium	2	0.4
New Zealand	3	0.3	Russia	2	0.4
Taiwan	3	0.3	Chile	2	0.4

### Number of studies and population

We assessed the relationship between the number of studies originating from each country and population size. Table 2 presents the number of studies published per one million inhabitants. Smaller countries generally made the greatest contribution, relative to size: Denmark with a population of 5.5 million published 3.82 studies per million inhabitants, followed by Sweden (9.0; 3.56), Finland (5.2; 3.27), the UK (61.1; 1.92) and Ireland (4.2; 1.66).

**Table 2:** Number of studies per million inhabitants per country within all specialities (left) and CAM-related studies (right).

Studies within all specialities			CAM-related studies		
Country	Population^1^	n/Pop^2^	Country	Population^1^	n/Pop^2^
Denmark	5.5	3.82	Denmark	5.5	2.00
Sweden	9.0	3.56	Finland	5.2	1.15
Finland	5.2	3.27	UK	61.1	0.97
UK	61.1	1.92	Sweden	9.0	0.89
Ireland	4.2	1.66	Norway	4.6	0.87
Australia	21.2	1.51	Israel	7.2	0.69
Netherlands	16.7	1.50	Netherlands	16.7	0.60
Israel	7.2	1.39	Hungary	9.9	0.50
Canada	33.4	1.38	Slovenia	2.0	0.50
Norway	4.6	1.30	New Zealand	4.2	0.48
Switzerland	7.6	1.05	Australia	21.2	0.47
USA	307.2	1.03	Switzerland	7.6	0.40
Austria	8.2	0.73	USA	307.2	0.37
New Zealand	4.2	0.71	Canada	33.4	0.30
Belgium	10.4	0.67	Austria	8.2	0.24
Gambia	1.7	0.60	Ireland	4.2	0.24
Italy	58.1	0.52	Italy	58.1	0.22
Slovenia	2.0	0.50	Germany	82.3	0.21
Greece	10.7	0.47	Belgium	10.4	0.19
Singapore	4.6	0.44	Taiwan	22.9	0.18
Germany	82.3	0.38	Libya	6.3	0.16
France	64.0	0.38	South Africa	19.0	0.16
South Africa	19.0	0.32	Ecuador	14.6	0.14
Hungary	9.9	0.30	Serbia	7.3	0.14
Spain	40.5	0.30	Romania	22.2	0.14
Czech Republic	10.2	0.29	Chile	16.6	0.12
Croatia	4.4	0.23	Poland	38.4	0.10
United Arab Emirates	4.7	0.21	Czech Republic	10.2	0.10
Portugal	10.2	0.20	France	64.0	0.09
Slovakia	5.4	0.19	Greece	10.7	0.09

1 Inhabitants in Million.

2 Number of studies/Million inhabitants.

When only CAM-related studies were considered, Denmark (5.5; 2.00) remained the leading country, followed by Finland (5.2; 1.15), UK (61.1; 0.97), Sweden (9.0; 0.89) and Norway (4.6; 0.87).

### Number of studies and spending on research & development

We also assessed the relationship between the scientific spending of the member states of the OECD and the number of studies published. The results are presented in table 3.

**Table 3:** Spending on research and development of each country and “studies for money” within all specialities (left) and CAM-related studies (right).

Studies within all specialities				CAM-related studies			
Country	GDP^1^	R&D^2^	n/R&D^3^	Country	GDP^1^	R&D^2^	n/R&D^3^
Denmark	196.3	2.54	4.21	Hungary	188.6	0.97	2.73
United Kingdom	2168.1	1.78^6^	3.03	Denmark	196.3	2.54	2.21
Greece	318.1	0.57	2.76	United Kingdom	2168.1	1.78^6^	1.53
Finland	183.6	3.47	2.67	New Zealand	114.8	1.16^5^	1.50
Sweden	334.8	3.63	2.63	Chile	199.8^5^	0.67^4^	1.49
Ireland	196.2	1.36	2.62	Slovenia	54.0	1.58	1.17
New Zealand	114.8	1.16^5^	2.25	Poland	609.4	0.56^6^	1.17
Netherlands	642.4	1.73	2.25	China	7055.1	1.49	1.07
Australia	794.6	2.01^6^	2.00	Norway	251.7	1.57	1.01
Slovak Republic	108.4	0.47	1.96	Finland	183.6	3.47	0.94
Canada	1269.6	1.89	1.92	Netherlands	642.4	1.73	0.90
Hungary	188.6	0.97	1.64	South Africa	463.3	0.95^6^ ^8^	0.68
Norway	251.7	1.57	1.52	Sweden	334.8	3.63	0.66
Italy	1802.2	1.14	1.46	Italy	1802.2	1.14^6^	0.63
South Africa	463.3	0.95^6^ ^8^	1.36	Australia	794.6	2.01^6^	0.63
Slovenia	54.0	1.58	1.17	Israel	188.9	4.74	0.56
Israel	188.9	4.74	1.12	Greece	318.1	0.57	0.55
Belgium	375.8	1.89	0.99	Canada	1269.6	1.89	0.42
Turkey	960.3	0.58^6^	0.90	India	3092.1	0.71^4^	0.41
Switzerland	308.6	2.9^4^	0.89	Ireland	196.2	1.36	0.37
United States	13741.6	2.68^7^	0.86	Switzerland	308.6	2.9^4^	0.34
Czech Republic	248	1.53	0.79	United States	13741.6	2.68^7^	0.31
Austria	308.7	2.56	0.76	Belgium	375.8	1.89	0.28
Chile	199.8^5^	0.67^4^	0.75	Czech Republic	248	1.53	0.26
Mexico	1479.9	0.46^5^	0.73	Austria	308.7	2.56	0.25
Spain	1417.4	1.2^6^	0.71	Germany	2829.1	2.53	0.24
Portugal	242	1.18	0.70	Turkey	960.3	0.58^6^	0.18
India	3092.1	0.71^4^	0.64	Spain	1417.4	1.2^6^	0.18
Poland	609.4	0.56^6^	0.59	Brazil	1833.6	1.02^6^	0.16
France	2078	2.08	0.56	France	2078	2.08	0.14

Latest available data were used. If not stated otherwise. data are of 2007.

1 Gross Domestic Product in billion US-Dollar;

2 Gross domestic expenditure on R&D;

3 Studies per billion US-Dollar spent on R&D;

4 2004;

5 2005;

6 2006;

7 R&D conducted by state and local governments is excluded;

8 Due to the lack of a comprehensive business register in South Africa. R&D expenditure may be underestimated by 10% to 15%.

Based on studies produced per billion US-Dollar of research and development spending, Denmark (4.21) achieved the highest productivity, followed by the UK (3.03), Greece (2.76), Finland (2.67) and Sweden (2.63).

When only CAM-related studies were considered, Hungary (2.73) was the leading country, followed by Denmark (2.21), the UK (1.53), New Zealand (1.50) and Chile (1.49).

## Discussion

### Summary of findings

The USA and the UK are major contributors to the worldwide pool of clinical studies. China plays an important role in the field of complementary and alternative medicine. However, in relation to size of population and spending on research and development smaller countries like Australia, Denmark, Finland, Ireland, New Zealand, and Sweden also play major roles.

Trial registers proved to be a poor source of additional information on the trials in our sample. Only a small proportion of trials were indexed in these databases. This reflects the fact, that the usage of trials registers was low until the statement of the International Committee of Medical Journal Editors (ICMJE) in 2004 [20], which increased the willingness for trial registration [21]; this effect is accentuated by the natural delay between conduct of trials and their inclusion in systematic reviews.

### Limitations and strengths

We decided to restrict our analysis to studies used for reviews conducted within the framework of the Cochrane Collaboration knowing that many other reviews are undertaken and published elsewhere [22]. These reviews could include studies not used in Cochrane Reviews. In our opinion, this should not limit the generalisability of our findings. Cochrane Reviews use robust, well documented searching methods and are likely to include a high proportion of the available studies in any clinical field [3], [23]. In addition, included studied underwent a rigorous quality assessment. Due to handsearching in various journals done by the Cochrane Collaboration, a number of non-English papers not included in other databases are part of the database searched for this project [23].

It should be noted that some clinical questions have been addressed by one or two high quality trials providing clear evidence in favour or against the use of interventions. In these cases systematic reviews might not have been undertaken. In our view, this should not influence the findings of this study as we used a large and representative sample of clinical studies.

Compared to total volume of published clinical studies worldwide, we included only small proportion of relevant studies. Due to the play of chance, our results might therefore be flawed. However, due to random sampling used for these studies systematic bias is unlikely.

In addition, we have decided to use the number of studies rather than the number of patients included in each study as this was not possible with the available data. While it could be argued that this might influence the findings presented in this paper (e.g. number of publications/population favours the production of smaller trials), we hope to have avoided measuring trial participation. However, it would be interesting for future studies to address this point and to allow comparisons between the two measures.

Our classification of likely country of origin was based on information given by numerous electronic databases and data given within the trial publications. In order to resolve uncertain cases we have used consensus. However, we acknowledge that even this consensus decision may be subjective and flawed. In addition, the accuracy of our decisions was linked to the quality of the trial publication. This may be independent of quality of the trial in whole.

We used gross domestic expenditures on research and development in general as a surrogate for spending in clinical research. This includes disciplines other than medical sciences and is therefore only an estimate of real spending on clinical research. However, in our opinion, this was the best way to ensure comparability of the different countries.

Furthermore, the data on R&D spending derived from the OECD database is partially outdated. Some of the stated information is older than other data used for table 3 of this paper. However, we do not believe that this variation is likely to have a major impact on the results.

To our knowledge, this is the first study to examine the production of clinical studies in the field of complementary and alternative medicine.

Additionally to previous publications, we linked the data on the number of publications in relation to national spending on research and development.

### Findings in context

We are able to confirm the main results of the study published by Gluud and Nikolova [15]. It is remarkable that smaller countries are able to publish more clinical studies than large countries with high GDP like China, France, Germany, Italy, Japan or Spain.

One could argue that language bias prevents publication by researchers from these countries in major international journals [24]. However, efforts of the Cochrane Collaboration lead to an inclusion of a substantial number of non-English language publications [23]. This argument is supported by the number of publications from China, which were identified in the area of the complementary and alternative medicine. However, this might be influenced by an increased activity and interest of Chinese researchers in the field of CAM.

### Implications

Despite the known differences in research funding between the USA and the European Union [25], there are differences between countries in Europe. Smaller countries like Denmark, Finland, Norway and The Netherlands produce more clinical studies than bigger countries like France, Germany, Italy and Spain. This could be due to a high proportion of R&D spending used for clinical research, a better organisation of research, or more focussed research programmes within these smaller countries. Significantly, discussion has started about a reform of countries' funding schemes [26].

### Conclusions

Our results support the leading roles of the USA and the UK in publishing clinical papers. The potential for a future important role for China can be seen when examining CAM studies. Taking into account size of population and economic power, countries like France, Germany, Italy, and Spain make small contributions. In contrast, smaller countries like Australia, Denmark, Finland, Ireland, New Zealand, and Sweden play major roles.

Research on factors explaining observed differences between countries could contribute to the design of future funding schemes and increase the efficiency of research spend.
